# Assessment of Awareness among Clinicians about Concepts in Undergraduate Pharmacology Curriculum: A Novel Cross-sectional Study

**DOI:** 10.4103/0975-1483.66797

**Published:** 2010

**Authors:** R Mahajan, NR Singh, J Singh, A Dixit, A Jain, A Gupta

**Affiliations:** *Department of Pharmacology, Adesh Institute of Medical Sciences and Research, Bathinda - 151 109, India*; 1 *Department of Pharmacology, Government Medical College, Amritsar - 143 001, India*; 2 *Department of Pharmacology, Sri Guru Ramdas Institute of Medical Sciences and Research, Amritsar - 143 001, India*; 3 *Department of Pharmacology, Maharishi Markandeshwar Institute of Medical Sciences and Research, Mullana - 133 203, Ambala, India*; 4 *Department of Pharmacology, Guru Gobind Singh Medical College, Faridkot - 151 203, India*; 5 *Department of Pharmacology, Gian Sagar Medical College and Hospital, Ram Nagar, Banur, Patiala, India*

**Keywords:** Clinicians, essential medicines, P-drugs, rational use of medicines

## Abstract

**Objective:**

In the last 30 years, concepts in pharmacology have moved from Essential Medicines (EM) to P-drugs via the Rational Use of Medicines (RUM), but no structured study has evaluated the level of understanding among working clinicians about these concepts. The present study is designed to fulfill that lacuna.

**Materials and Methods:**

A cross-sectional study was carried out in and around the teaching hospitals attached to Medical Colleges, enrolling 504 clinicians from six centers across North India to fill-up a questionnaire containing 25 questions. The results were compiled using percentages and averages.

**Results:**

Only one-fourth of the participants claimed that they always prescribed Essential Medicines; no one could accurately count the number of drugs / drug combinations in the Indian Essential Drug list; only 15.1% of the clinicians used to write the generic names of drugs on a prescription slip; about one-third of the clinicians were not fully aware about the adverse effects, drug interactions, and contraindications of the drugs they prescribed; about 83% of the physicians admitted to rely on information from Medical Representatives (MRs) and the interest in research activities seemed to be lost.

**Conclusion:**

Results show a sorry state of affairs among clinicians, as far as the level of understanding about EM, P-drugs, and RUM is concerned, and points toward arranging more continuing medical education (CME) for clinicians regarding these concepts.

## INTRODUCTION

The first World Health Organization’s (WHO) Model List of Essential Medicines was drawn up in 1977, in response to a request from the World Health Assembly, and since then this Model List has been revised and updated continuously.[1] In the last 30 years, we have moved from Essential Medicines (EM) to Personal drugs (P-drugs) via Rational use of Medicine (RUM) and Evidence-Based Medicine.

The selection of essential medicines is only one step toward the improvement of the quality of healthcare; the selection needs to be followed by appropriate use. Each individual should receive the right medicine, in an adequate dose, for an adequate duration, with appropriate information and follow-up treatment, and at an affordable cost.[1] This forms the corner-stone of the concept of Rational Use of Medicine.

To boost the cause of RUM, the P-drug concept was given in the late nineties. The idea was to make physicians familiar with few Personal drugs chosen from national essential drug list based on efficacy, safety, suitability, and cost, with regard to the population they cater.[2] The WHO has developed recommendations for twelve core national policies and structures that are needed to promote the rational use of medicines. The main areas where developing countries are still lagging behind are problem-based pharmacotherapy training in the undergraduate curriculum, continuing in-service medical education as a licensure requirement, independent information on medicines, and avoidance of perverse financial incentives.[3]

Problem-based pharmacotherapy training is not a part of undergraduate training in most of the developing countries. Although, a debate has now started, to include it in undergraduate training,[4,5] and it is being tested in some medical schools,[6,7] but the professionals who are already in practice hardly know these concepts. Moreover, in-service continuing medical education (CME) is lacking in developing countries; and if at all CME is conducted, it is mostly sponsored by drug houses having their own market interests. More importantly, physicians rely on drug information provided by medical representatives (MRs).

In the light of the aforementioned discussion, the present study was conducted to assess the awareness of the concepts of RUM, EM, P-drugs, and sources of drug-information, among clinical practitioners across North India.

## MATERIALS AND METHODS

A cross-sectional study was conducted simultaneously in and around six referral teaching hospitals attached to Medical Colleges across North India, in February 2010, after taking approval from the Institutional Ethical Committees. Clinicians from different disciplines working in these Medical Colleges and also clinicians working in the vicinity of these Medical Colleges, having degrees in the allopathic system of medicine and practicing in the allopathic system of medicine, who gave a consent to participate in the study, were given a pre-formed questionnaire to be filled in the presence of an investigator at that particular time, just to ensure that nobody got a chance to consult books or other relevant materials. An undertaking was given not to use any data subjectively or to disclose the identity in any other way. To widen the scope of the study, clinicians working in the Dentistry Departments of Medical Colleges, House Officers (MBBS), and General Duty Medical Officers (GDMOs) were also included in the study, but doctors working in pre- and para-clinical disciplines were excluded from the study.

In all, the questionnaire had 25 questions divided into five sections. Section-A dealt with personal information of physicians, such as, name, address of work place, highest academic qualification, and field of specialization. Section-B dealt with Essential Medicine; Section-C with Rational use of Medicine; Section-D with concept of P-drugs; and Section-E dealt with sources of information. At the end of the study, all the data was pooled and results were analyzed in percentages and averages.

## RESULTS

In all, 650 clinicians were contacted. Of these 650 clinicians, 82 clinicians refused to give consent, 34 clinicians were registered medical practitioners without a valid degree in any system of medicine, 24 clinicians were practicing in the allopathic system of medicine, but had degrees in the Indian system of medicine, and six clinicians had degrees in the allopathic system, but were practicing homeopathy. Thus a total of 504 fully filled and valid questionnaires were received. Out of these 504 respondents, 280 (55.6%) were males and 224 (44.4%) were females. The age of the study participants ranged from 24 to 61 years. A majority of respondents had postgraduate degrees (80.2%); 200 had MD degrees, 152 were MS, 44 were MDS, and eight were diploma holders. Out of 84 graduates, 68 were MBBS and 16 were BDS. [Table 1].

**Table 1:** Background characteristics of responders

Characteristics	(n = 504) N (%)
Age (years)	
< 40	324 (64.3)
≥ 40	180 (35.7)
Sex	
Males	280 (55.6)
Females	224 (44.4)
Educational qualification	
Graduates	84 (16.7)
Postgraduates	404 (80.2)
DM / MCh	16 (3.1)
Total experience (years)	
< 10	340 (67.5)
≥ 10	164 (32.5)

Fourteen of the total 25 questions had answers either in yes or no / right or wrong format. The responses of physicians to these questions are shown in Table 2. As is clear from the data, although 84.9% of the physicians claimed to take care to prescribe an essential medicine, only 46.8% of physicians were aware about the fact that the new term used now was ‘essential medicines’ instead of ‘essential drugs’. When those physicians who claimed to prescribe essential medicines were further questioned, only one-fourth of the physicians stated that they always prescribed essential medicines [Table 3]; 28.6% of the physicians had the National Model Essential Drug List (EDL) available at their work place, but ironically none of the participants were aware about the exact number of drugs / drug combinations included in the National Model EDL. Only two clinicians (0.4%) were able to correctly name the various parts of the prescription slip.

**Table 2:** Response of Physicians to yes or no / right or wrong questionnaire

Question / Statement	Response (Yes / Right, n = 504) N (%)
Are you aware of the term essential drugs?	440 (87.3)
Are you aware of the term used is essential medicines now?	236 (46.8)
Do you take care to prescribe an essential medicine?	428 (84.9)
Do you have National Model EDL at your work place?	144 (28.6)
How many drugs are included in the Indian EDL?	0 (0)*
How many drug combinations are included in the Indian EDL?	0 (0)*
Are you aware of the term rational use of medicine?	428 (84.9)
If yes, do you practice rational use of medicine?	420 (83.3)
Can you name the parts of a prescription?	52 (10.3)
If yes, name the parts of a prescription	2 (0.4)*
Are you aware of the term P-drugs?	116 (23.0)
If yes, do you practice it (concept of P-drugs)?	100 (19.8)
Do you always have full knowledge of the ingredients of the medication you prescribe?	360 (71.4)
Are you always aware of the AEs, interactions and contraindications of the drugs you prescribe?	344 (68.3)

EDL: Essential drug list, AEs: Adverse effects, Participants responses were recorded as Yes or No / Right* or Wrong. Table indicates number (%) with Yes / Right answers only.

**Table 3:** Response to multiple choice questions

Question / Statement	Response N (%)
How often do you prescribe an essential drug?	Always 108 (25.2*)
Frequently	248 (57.9*)
Occasionally	72 (16.8*)
What do you prefer to write on a prescription slip?	Generic name 76 (15.1)
Trade name	320 (63.5)
Both	108 (21.4)
What do you prefer to prescribe, a new or old drug?	New drug 64 (12.7)
Old drug	156 (31.0)
Both	284 (56.3)

* Percentage of physicians who claimed to prescribe essential drugs (n = 428), otherwise n = 504

Three structured questions had more than two options to answer from, and responses to these questions are shown in Table 3. Despite a large claim by clinicians that they practiced rational use of medicine (83.3%); only 15.1% of the clinicians wrote the generic name of drugs on the prescription slip, while a large number (63.2%) wrote the trade name of the drugs [Table 3]. Moreover, about one-third of the clinicians were not fully aware of the adverse effects, drug interactions, and contraindications of the drugs they prescribed [Table 2].

The remaining eight questions dealt with the sources of information, participation in CMEs / conferences, and interest in publishing articles. Here, the responses needed to be quantified, as shown in Table 4. Physicians were asked to name all the sources from where they collected drug-information. About 83% of the physicians admitted to rely on information from MRs, while 69% used the internet also. The average number of journals prescribed individually by physicians was only 0.6 per individual. Similarly, the number of presentations and publications during the last one year was only 0.2 and 0.1 per individual, respectively.

**Table 4:** Quantified responses of participants about source of information

Question / Statement	Response (n = 504) N (% or Average)
Sources of primary drug information	
Review articles in journals	348 (69.0)
Referring drug indices	276 (54.8)
Referring standard text books	216 (42.9)
Information from medical representatives	420 (83.3)
Checking on internet	348 (69.0)
Number of journals subscribed individually	312 (0.6*)
Number of CMEs / conferences attended in last one year	384 (0.8*)
Number of CMEs / conferences attended in life	2132 (4.2*)
Number of articles / posters presented in last one year	96 (0.2*)
Number of articles / posters presented in life	720 (1.4*)
Number of articles published in last one year	32 (0.1*)
Number of articles published in life	584 (1.2*)

* Average, otherwise percentage

## DISCUSSION

Earlier, studies have been conducted to assess the understanding levels of students regarding problem-based learning,[6] P-drug concept,[7] and computer-assisted learning (CAL).[8,9] This study is unique and novel in the sense that no effort has been made earlier to assess the level of understanding among working clinicians, regarding prevalent concepts in pharmacology, namely, Essential Medicines, Rational Use of Medicine, and P-drugs.

The Essential drug concept is an old concept, and over 30 years have passed since it was first mooted. A majority of physicians were aware of Essential drugs; but less than half (46.8%) were aware of the new term ‘Essential Medicine’. Moreover, only one-fourth of the physicians always cared to prescribe an essential drug. Ironically, no physician was able to quantify the drug / drug combinations in the Indian EDL accurately. This clearly indicates the lack of continued medical education.

Despite the fact that 83.3% clinicians claimed to practice RUM, only 15.1% of the physicians wrote the generic name of the drug, only two could write parts of a prescription correctly, only 71.4% of the clinicians had complete knowledge of the ingredients of the medicament they had prescribed, and about 32% physicians were not fully aware about the adverse effects, interactions, and contraindications of the drugs they were prescribing. Moreover, there was a heavy reliability and dependence on MRs for drug information. Time and again, all these factors have been labeled as lures promoting the irrational use of medicines.

The P-drug is quite a new concept, and in India, this concept has started gaining importance only in the last two to three years.[10,11] As a result, only 23% of the physicians were aware about the P-drug concept and only one-fifth claimed to practice it, clearly indicating the need to arrange more CME on this issue.

Research orientation among physicians working in teaching hospitals seemed to be lost. The individual subscription rate for journals was only 0.6 per individual; while the number of CME programs attended during the last one year was only 0.8 per individual. On an average, each physician presented only 0.2 posters / articles and published 0.1 articles during the last one year. The loss of interest in research activities could be due to the fact that in the earlier teaching eligibility criteria, as prescribed by Medical Council of India (MCI), requirement of research publications was only a desirable qualification for promotions to higher ranks.[12] However, with the implementation of the new amendments from August 2009, the requirement of research publications has been made mandatory by the MCI.[13] Thus, hopefully conditions will improve.

## CONCLUSION

Practicing what we teach remains a big challenge.[14] Prescribing drugs by using their trade names, prescribing new drugs more commonly, and dependence on MRs for medical information by physicians points to clear-cut favoritism toward market driven forces; ultimately leading to an irrational use of medicines. Lack of knowledge about the P-drug concept, present EDL, and RUM, underlines the need for arranging continued in-service medical education programs, on basic pharmacological concepts, for physicians. The importance of introducing these concepts in the undergraduate curriculum is unquestionable, but, developing new concepts without their field utilization is fruitless. It is urgently required that these concepts be liberated from classroom custody and be implemented in pragmatic and field situations.

## RECOMMENDATIONS BY AUTHORS

Many of the present pharmacological concepts related to undergraduate curriculum were recently incorporated. Obviously the positive-response rate of the physicians regarding these newer concepts (P-drugs, RUM) was poor. In order to promote the rational use of medicine and to further the cause of these pharmacological concepts in real world use, the following recommendations / suggestions may hold good:

More in-service CMEs should be arranged for practicing physicians regarding these concepts of undergraduate pharmacological curriculum; particularly for those who had left the Medical Colleges before these concepts came into existence.

Earning fixed number of CME credit hours per year should be made mandatory for renewal of registration with medical councils (already MCI and some state councils, such as the Punjab Medical Council, has made it a pre-requisite for license renewal).

Government should try to have authenticated and up-to-date drug centers to cater round the clock demand of physicians; so that dependence on MRs is lessened (One such center is working in the Department of Pharmacology, PGIMER, Chandigarh, in North India).

Physicians should be encouraged to use old and time-tested drugs more often, where applicable; as field experience with these drugs is immense.

Use of internet and information technology techniques in medical education should be encouraged.
